# Frequent migration of introduced cucurbit-infecting begomoviruses among Middle Eastern countries

**DOI:** 10.1186/1743-422X-11-181

**Published:** 2014-10-09

**Authors:** Moshe Lapidot, Dana Gelbart, Amit Gal-On, Noa Sela, Ghandi Anfoka, Fatima Haj Ahmed, Yusuf Abou-Jawada, Hana Sobh, Hamed Mazyad, Aboul-Ata E Aboul-Ata, Ahmed Kamal El-Attar, Mohammed S Ali-Shtayeh, Rana M Jamous, Jane E Polston, Siobain Duffy

**Affiliations:** Institute of Plant Sciences, Volcani Center, P.O. Box 6, Bet Dagan, 50250 Israel; Institute of Plant Protection, Agricultural Research Organization, Volcani Center, P.O. Box 6, Bet Dagan, 50250 Israel; Department of Biotechnology, Al-Balqa’ Applied University, Al-Salt, 19117 Jordan; Plant Pathology Research Institute, ARC, P.O. Box 12619, Giza, Egypt; Biodiversity and Biotechnology Research Unit, Biodiversity & Environmental Research Center (BERC), P.O.B. 696, Til-Nablus, Palestine; Department of Plant Pathology, University of Florida, Gainesville, FL 32611 USA; Department of Ecology, Evolution and Natural Resources, Rutgers, the State University of New Jersey, New Brunswick, NJ 08901 USA

## Abstract

**Background:**

In the early 2000s, two cucurbit-infecting begomoviruses were introduced into the eastern Mediterranean basin: the Old World *Squash leaf curl virus* (SLCV) and the New World *Watermelon chlorotic stunt virus* (WmCSV). These viruses have been emerging in parallel over the last decade in Egypt, Israel, Jordan, Lebanon and Palestine.

**Methods:**

We explored this unique situation by assessing the diversity and biogeography of the DNA-A component of SLCV and WmCSV in these five countries.

**Results:**

There was fairly low sequence variation in both begomovirus species (SLCV π = 0.0077; WmCSV π = 0.0066). Both viruses may have been introduced only once into the eastern Mediterranean basin, but once established, these viruses readily moved across country boundaries. SLCV has been introduced at least twice into each of all five countries based on the absence of monophyletic clades. Similarly, WmCSV has been introduced multiple times into Jordan, Israel and Palestine.

**Conclusions:**

We predict that uncontrolled movement of whiteflies among countries in this region will continue to cause SLCV and WmCSV migration, preventing strong genetic differentiation of these viruses among these countries.

## Background

The frequent movement of seeds and vegetative plant material across national borders is known to contribute to the spread of plant diseases [1]. Plant diseases that are vectored by arthropods can also move where their vectors move, from field to field, and between adjacent countries. Despite the economic importance of maintaining phytosecurity, the movement of plant pathogens between countries is poorly studied [2]. We usually do not know whether the isolates of emerging plant pathogens within a region are part of a single, cohesive population on a genetic level, or if they reflect geographic structuring, nor do we know if the pathogen was introduced once or multiple times into the region (e.g., [3]).

Since the turn of the millennium, two new viruses have emerged in cucurbits in the eastern Mediterranean basin. Both are bipartite begomoviruses: single-stranded DNA viruses transmitted by members of the *Bemisia tabaci* species complex. *Squash leaf curl virus* (SLCV) was first observed in California in the late 1970’s, and has subsequently been well-characterized [4]. Its arrival in Israel marked the first time a New World begomovirus was found causing disease in the Old World [5, 6]. *Watermelon chlorotic stunt virus* (WmCSV) is an Old World virus, originally isolated in Yemen in the Arabian peninsula [7], but not observed prior to 2002 in Egypt, Israel, Jordan, Lebanon and the portions of Palestine governed by the Palestinian Authority (in the West Bank). Both of these migrants rapidly spread among the countries of the Middle East. SLCV was first noticed in Israel in 2002 and became epidemic there in 2003 [5.6]. By 2005 it had spread to Egypt [8], Jordan [9], and then was isolated from Lebanon [10] and Palestine [11, 12] in 2008. The WmCSV epidemic ballooned from a single symptomatic watermelon plot in southern Israel in 2002 [6] to affect cucurbit production in Lebanon in 2009 [13], Palestine in 2010 [14] and Jordan in 2011 [15]. These viruses produce more severe symptoms when they co-infect the same plant [16], making their spread even more significant for agriculture in the Middle East.

These contemporaneous emergences afford the unique opportunity to compare the population dynamics of two begomoviruses that use some of the same hosts and vectors in the same region at the same time. SLCV has established itself thousands of miles away from its relatives, while WmCSV moved a much shorter distance, within the Old World. Despite their different journeys, our results show that both viruses may have entered the region only once, are similarly diverse, and the gene flow among nations in the Mediterranean basin is similar for both SLCV and WmCSV.

## Results

**Figure 1:**
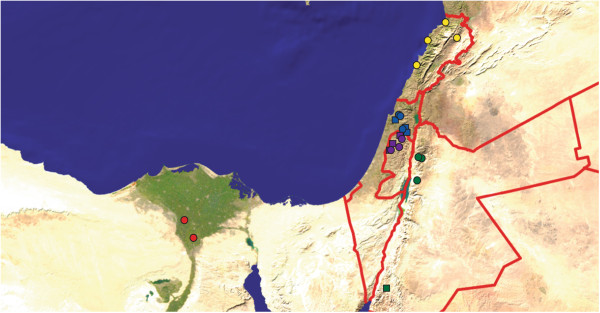
**Sampling locations.** Squash fields sampled are shown as circles, and watermelon fields as squares. Sites in Egypt are shown in shades of red, in Israel in shades of blue, Jordan in shades of green, Lebanon in shades of yellow and in Palestine in shades of purple. The background image is from primap.com, which grants unrestricted permission for noncommercial use.

Sequences of SLCV were obtained from all five countries in this study (Table 1, Figure 1), but sequences of WmCSV were only obtained from three (Table 2, Figure 1). Although WmCSV has been found previously in Lebanon [13], no symptomatic watermelons were observed there during the sampling times. WmCSV has not yet been reported in Egypt. Sampling sites were located at least 9 km apart, with one exception: as WmCSV was only found in one location in Jordan, its three sampling sites were less than half a kilometer apart.

**Table 1:** **Symptomatic squash samples collected**

Country	Site/region	Site	Coordinates	No. of fields	Total no. samples	No. SLCV positive
Egypt	Menoufia	1	30°33'57.2"N 31°00'47.2"E	7	50	15
	Ismallia/Qalyoubia	2	30°17'29.8"N 31°11'53.2"E	8	267	97
Israel	Sandala	1	32°31'21.5"N 35°19'04.9"E	2	30	16
	Kafr Manda North	2	32°48'56.9"N 35°14'51.1"E	2	40	33
	Kafr Manda South	3	32^o^48’11.0”N 35^o^15’43.7”E	2	34	34
Jordan	Madaba	1	31°43'10.3"N 35°47'38.8"E	6	120	120
	Baqa’	2	32°03'27.7"N 35°45'57.7"E	3	42	42
	Sero’	3	32°01'13.5"N 35°50'36.5"E	2	25	25
Lebanon	Baalbek-Hermel	1	34°12'29"N 36°17'59"E	1	11	5
	Amsheet	2	34°09'01.4"N 35°38'09.5"E	1	8	8
	Akkar	3	34°29'58.3"N 35°59'38.7"E	5	24	24
	Jiyeh	4	33°40'24.1"N 35°25'30.5"E	3	14	14
Palestine	Jenin	1	32°26'42.0"N 35°19'01.2"E	2	70	60
	Til/Nablus	2	32°11'45.6"N 35°12'03.6"E	2	31	19
	Qalaqilia	3	32°11'09.6"N 34°59'31.2"E	2	38	37

**Table 2:** **Symptomatic watermelon samples collected**

Country	Site/region	Site	Coordinates	No. of fields	Total no. samples	No. WmCSV positive
Israel	Newe Ya’ar	1	32°42'40.9"N 35°09'57.1"E	1	18	18
	Nir David	2	32°30'02.1"N 35°26'48.3"E	2	31	24
	Geva	3	32°33'58.0"N 35°21'59.3"E	1	20	12
Jordan*	Ghor Al-Safi	1-3	29°48'14.6"N 35°18'29.7"E	6	105	105
Palestine	Jenin	1	32°27'14.4"N 35°18'57.6"E	2	50	23
	Qalaqilia	2	32°11'16.8"N 34°58'51.6"E	2	50	22
	Tulkarem	3	32°18'39.6"N 35°02'52.8"E	3	62	0

*WmCSV was only found in one region, and the three sites were in north (site 1), central (site 2) and south (site 3) of Ghor Al-Safi. The sites were 200-300 meters apart, and two fields were sampled in each site.

A total of 149 SLCV (136 haplotypes) and 106 WmCSV (93 haplotypes) DNA-A genome sequences were analyzed in separate datasets (Table 3). Both viral data sets were not particularly diverse (nucleotide diversity, π = 0.0077 for SLCV, π = 0.0066 for WmCSV, Table 3). Very few putative recombinants were identified in the SLCV dataset and none in the WmCSV data set (Table 4).

**Table 3:** **Population diversity and structure within SLCV and WmCSV samples in this study**

	Collection site	SLCV			WmCSV		
		Number of sequences	π	F _ST_	Number of sequences	π	F _ST_
**Egypt**		**15**	**0.0093**	**0.0638**			
	1	7	0.012				
	2	8	0.0060				
**Israel**		**38**	**0.0081**	**0.0146**	**31**	**0.0066**	**0.183**
	1	13	0.012		9	0.0064	
	2	12	0.0056		11	0.0043	
	3	13	0.0061		11	0.0064	
**Jordan**		**59**	**0.0040**	0*	**56**	**0.0058**	**0.311**
	1	20	0.0042		17	0.0018	
	2	20	0.0041		19	0.0060	
	3	19	0.0042		20	0.0054	
**Lebanon**		**11**	**0.0122**	**0.0193**			
	1	1					
	2	3	0.012				
	3	6	0.012				
	4	1					
**Palestine**		**26**	**0.0047**	**0.116**	**19**	**0.0055**	**0.462**
	1	10	0.0053		10	0.0029	
	2	10	0.0029		9	0.0048	
	3	6	0.0048				
**All**		**149**	**0.0077** ± 0.00077	**0.253**	**106**	**0.0066** ± 0.00019	**0.151**

Fst is the fixation index, π is per-site, pairwise nucleotide diversity.

*This Fst value was negative, rounded to zero.

**Table 4:** **Putative recombinants in the SLCV dataset detected by up to seven algorithms in RDP 3.44**

Isolate	Region	Major parent	Minor parent	Identified by
IL1-1	1442-1812	IL2-53	Unknown	GBMCS3
LB3-6-41	1002-1603	IL3-99	Unknown	RGBMCS3
LB2-2-12	1296-1458	LB1-1-8	Unknown	GMCS3
IL2-57	1661-1932	IL1-27	Unknown	MCS3

RDP (R), Geneconv (G), Bootscan (B), Maximum Chi Square (M), Chimaera (C), SisterScan (S) and 3Seq (3) methods implemented in Recombination Detection Program.

Within each country, viruses were isolated from multiple locations. This facilitates studying geographic structuring within each species. SLCV showed very low levels of genetic differentiation between sample sites within each country, indicating frequent migration of viruses between sites within a single country (Table 3). This was especially true within Jordan, where no differentiation by site of isolation was observed. The barriers to SLCV migration are apparently larger between countries, as evidenced by a higher fixation index (Table 3). WmCSV showed a much more geographically structured distribution than SLCV. Within each country there was more evidence for viruses at the same site being more similar to each other than to those at other sites. However, the barriers to migration among the neighboring countries of Israel, Jordan and Palestine are low, and overall there was more evidence for migration of WmCSV across borders than for SLCV (SLCV F_ST_ = 0.253 compared to WmCSV F_ST_ = 0.151, Table 3). The homogeneity of WmCSV is all the more surprising, given the substantial distance between the WmCSV sampling sites in Israel and Palestine and sampling sites in southern Jordan (Figure 1).

**Figure 2:**
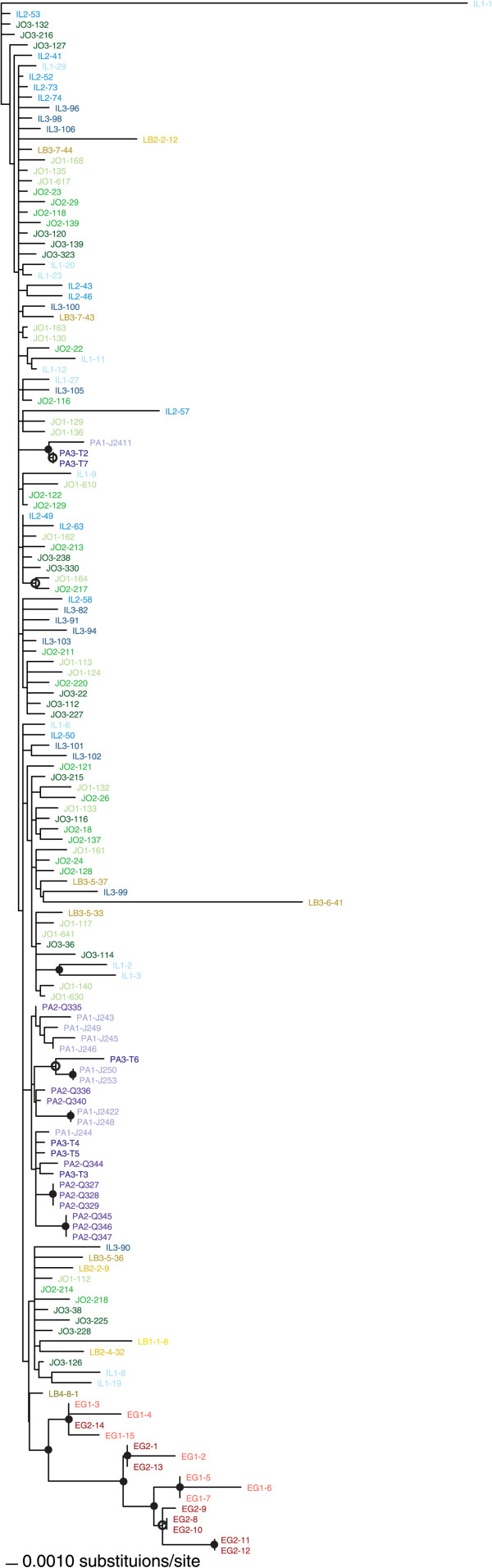
Maximum likelihood unrooted phylogeny of SLCV DNA-A isolated in this study, created in PAUP* using a general time reversible nucleotide substitution model with a gamma distribution of site heterogeneity. Sequences from Egypt (EG) are shown in shades of red, from Israel (IL) in shades of blue, Jordan (JO) in shades of green, Lebanon (LB) in shades of yellow and the regions of Palestine governed by the Palestinian Authority (PA) in shades of purple. Sequences from a common site were shown in the same shade, up to four sites per country. Bootstrap support of ≥85% from both PAUP* and RaxML trees is shown by a solid circle; an open circle denotes ≥85% support from only RaxML.

The biogeography of SLCV sequences is visible in their maximum likelihood (ML) phylogenetic relationships (Figure 2). SLCV sequences show some clustering by country, most notably the Egyptian isolates forming a well-supported clade. Isolates from Palestine largely grouped together, but without the same bootstrap support. Isolates from Israel, Jordan and Lebanon were thoroughly intermingled. There was no pattern of clustering based on site of isolation, consistent with the low F_ST_ values in Table 3. The longest branch on the tree belonged to Israeli isolate IL1-1, which has a large deletion (in Rep: starting around residue 209, and ending just before the overlapping reading frame for AC2, encompassing most of the nuclease domain) compared to the other sequences (2356 nt, instead of the more typical >2600 for the DNA-A segments of bipartite begomoviruses).

Looking past national borders, some of the sampling sites in different countries were fairly close together (Figure 1). There was an overall trend of SLCV sequences being more genetically similar when sampled closer together (r = 0.41, p = 0.0001). However, this correlation was driven by the monophyletic, divergent Egyptian sequences, which were sampled at least 428 km away from sites in other countries. When only the sequences from Israel, Jordan, Lebanon and Palestine were considered there was a dramatic drop in the relationship between geographic and genetic distance, though it was still statistically significant (r = 0.15, p = 0.043). This result quantifies the qualitative intermingling of isolates from these nations in the phylogenetic tree (Figure 2).

**Figure 3:**
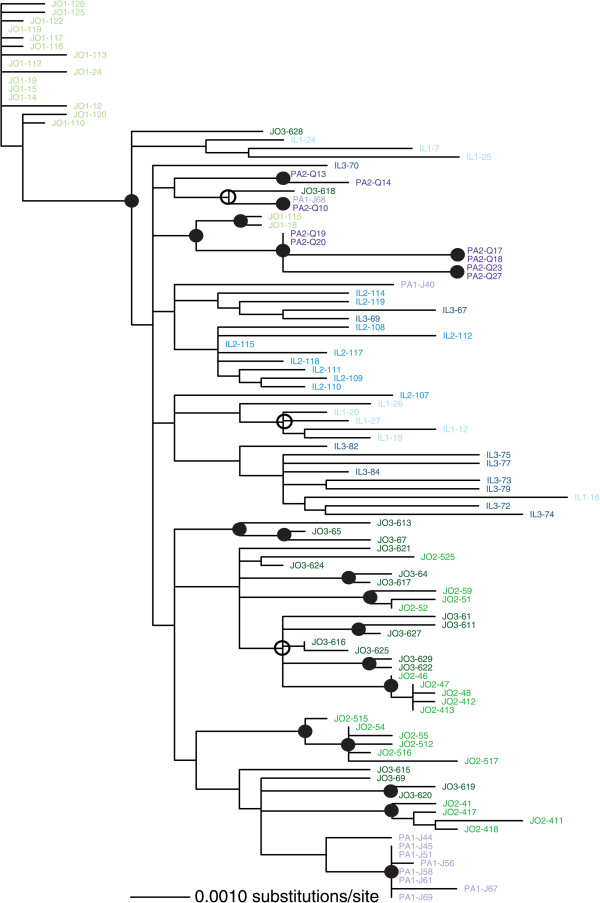
Maximum likelihood unrooted phylogeny of WmCSV DNA-A isolated in this study, created in PAUP* using a transitional nucleotide substitution model with a gamma distribution of site heterogeneity. Sequences from Israel (IL) in shades of blue, Jordan (JO) in shades of green, and the regions of Palestine governed by the Palestinian Authority (PA) in shades of purple. No WmCSV samples were isolated in Egypt and Lebanon during this study. Sequences from a common site were shown in the same shade, up to three sites per country. Bootstrap support of ≥85% from both PAUP* and RaxML trees is shown by a solid circle; an open circle denotes ≥85% support from only RaxML.

The biogeography of the WmCSV sequences (Figure 3) showed a clustering pattern similar to that predicted by the results in Table 3. Some of the well-supported groupings contained sequences from only a single isolation site; most notably, most of the sequences from site 1 in Jordan formed a clade. The sequences were again fairly well mixed among the countries of isolation, consistent with migration among these nearby nations (Figure 3). The relationship between sampling location and genetic distance was again significant (r = 0.24, p = 0.0001), but it was not as strong as it was for the full SLCV data set.

**Figure 4:**
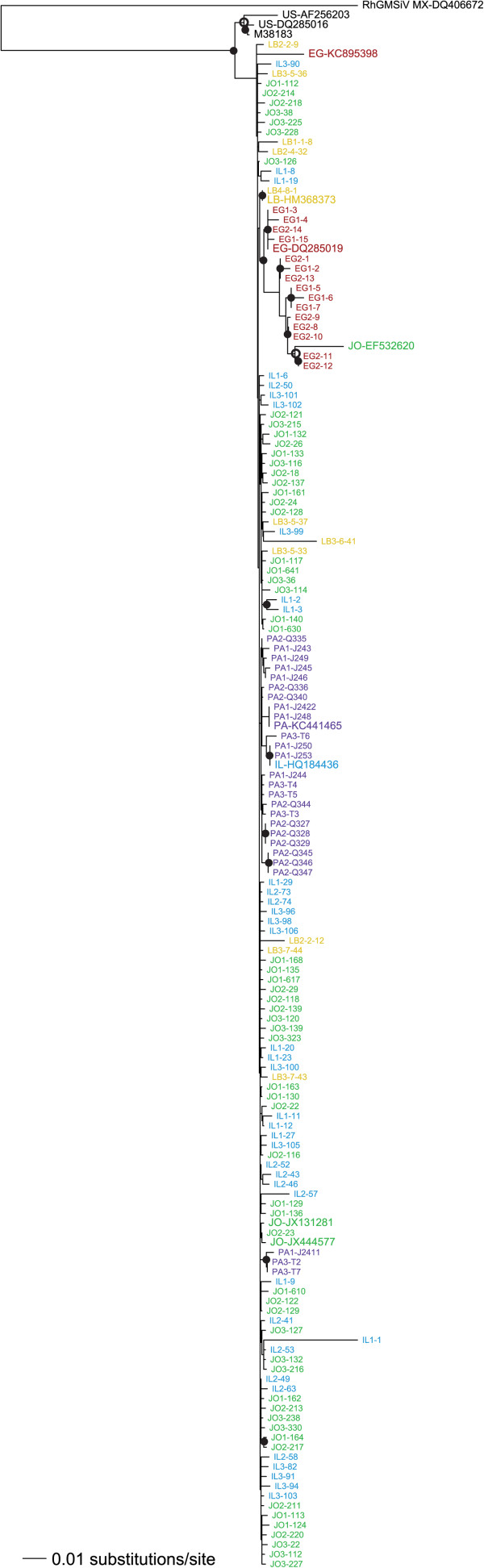
Maximum likelihood midpoint-rooted phylogeny of SLCV DNA-A both isolated in this study and from GenBank, created in PAUP* using a Tamura-Nei nucleotide substitution model with a gamma distribution of site heterogeneity. The locations from which various GenBank sequences were isolated, where available, were obtained from the GenBank file or from the associated publications. Sequences from Egypt (EG) are shown in red, from Israel (IL) in blue, Jordan (JO) in green, Lebanon (LB) in yellow and the regions of Palestine governed by the Palestinian Authority (PA) in purple. Sequences from all other countries are shown in black, with their two-letter country code preceding the accession number. Bootstrap support of ≥85% from both PAUP* and RaxML trees is shown by a solid circle; an open circle denotes ≥85% support from only RaxML. The closely related *Rhynchosia golden mosaic Sinaloa virus* (RhMSV) is also included, and serves as an outgroup.

We then combined our datasets with whole DNA-A sequences available in GenBank. ML phylogenies including both isolates from this study and those isolated previously, or in other countries, are shown in Figures 3 and 4. SLCV in the Middle East is reciprocally monophyletic with isolates from the New World, supporting a single introduction into the Old World (Figure 4). The additional GenBank sequences showed that more migration events have occurred than were found in our survey, for instance, a Jordanian isolate (EF532620) now nests within the previously all-Egyptian clade. For another example, an Egyptian sequence from GenBank (KC895398) did not group with the other Egyptian isolates. A Shimodaira-Hasegawa (SH) test indicated that this likely reflects two separate migrations of SLCV into Egypt: the ML tree in Figure 4 is a significantly better fit to the dataset than one that forces all the Egyptian isolates to be in a single clade, p = 0.013. We employed an identical test to see if the two groupings of SLCV from Palestine could be explained by one single migration event. The SH test results strongly rejected that in favor of the two introductions implied by Figure 4 (p = 0.009). Our analysis supports a single introduction of SLCV into the eastern Mediterranean basin from the New World, but none of the five countries studied had SLCV isolates originating from a single introduction of the pathogen.

**Figure 5:**
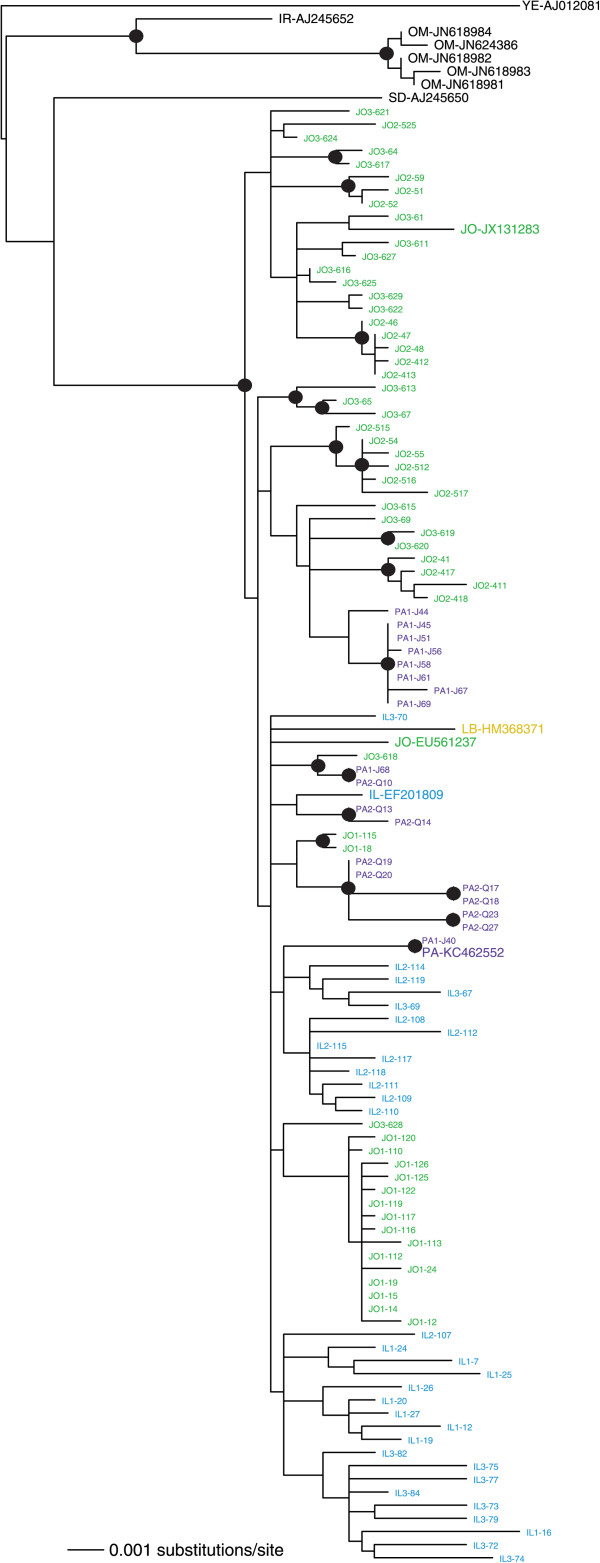
Maximum likelihood midpoint-rooted phylogeny of WmCSV DNA-A both isolated in this study and from GenBank, created in PAUP* using a Kimura two-parameter nucleotide substitution model with a gamma distribution of site heterogeneity and a parameter for the proportion of invariant sites. The locations from which various GenBank sequences were isolated, where available, were obtained from the GenBank file or from the associated publications. Sequences from Israel (IL) are shown in blue, Jordan (JO) in green, Lebanon (LB) in yellow and the regions of Palestine governed by the Palestinian Authority (PA) in purple. Sequences from all other countries are shown in black, with their two-letter country code preceding the accession number. Bootstrap support of ≥85% from both PAUP* and RaxML is shown by a solid circle.

WmCSV was introduced into these five countries from a source much closer than that of SLCV. WmCSV is an Old World bipartite begomovirus that has been isolated in Iran, Sudan, Yemen and Oman [17, 18]. Isolates in our study were found to be most closely related to a WmCSV sequence from Sudan, although without strong bootstrap support (Figure 5). The isolates from the studied countries were monophyletic with respect to these other countries, but as was the case with SLCV among Israel, Jordan, Lebanon and Palestine, the sequences isolated in these four countries showed no particular geographic structure.

## Discussion

While pathogen movement between the Old and New World is on the rise [19], there have been very few instances where a begomovirus from one part of the world becomes a successful ‘invasive species’ in another. To date, SLCV is the only case of a New World virus establishing in the Old World [8], and *Tomato yellow leaf curl virus* would be the only example of the reverse [20]. In this survey, we compared the emergence of the New World SLCV with the Old World WmCSV. Our results showed remarkable similarity between these two viruses, indicating that a New World virus does not face any particular hurdles when emerging in the Old World.

The low sequence variation observed is consistent with the low levels of recombination detected in the data set. Interspecific recombination increases a population’s average pairwise nucleotide differences (π) [21]. The nucleotide diversity values are similar to some previous studies that quantified intraspecific sequence variation of *Tomato severe rugose virus* and *Tomato yellow vein streak virus* within Brazil, over regions comparable to the size sampled in this study [21, 22]. However, the values are lower than those measured for many plant virus species, [23, 24], and these two Brazilian begomoviruses also showed low levels of detectable recombination. The Brazilian begomoviruses are themselves newly recognized and emergent after a host shift into tomato crops, which could mean that emergent begomoviruses are less diverse than their more well-established counterparts. There are no appropriate datasets of SLCV variation in the Americas or WmCSV in the eastern Middle East with which to compare and evaluate whether emergent viruses have smaller amounts of sequence variation. Arguing against this possibility is the fact that begomoviruses are able to diversify rapidly [25]. Nonetheless, this remains an intriguing possibility, and calls for population diversity surveys over time in the same locations.

Some of the isolates sequenced had premature stop codons or alternative start codons for some of the overlapping reading frame genes. While there has been some characterization of aberrant isolates [6], overall we are not certain whether some of these genomes would be capable of replicating and causing infection on their own. These sequences may not be mere sequencing errors since complementation can occur during begomovirus infection [26]. For instance, most Egyptian SLCV isolates shared a common premature stop codon at residue 122 in Rep (nine of 15 total isolates, split between the two isolation sites), which indicates that this mutation is circulating at high frequency in Egyptian fields.

The major difference between the two viruses was in population substructure. SLCV and WmCSV have been in the region for roughly the same length of time, but SLCV isolates are homogenous over sites within the same country. Different sites were used for the SLCV and WmCSV samplings, and increased differentiation among WmCSV sampling sites would imply increased distance between them. However, the WmCSV sampling sites within each country were actually closer together than the SLCV sites were (Tables 1 and 2), and WmCSV had an overall low correlation between genetic and geographic distance. It is worth noting that the three Jordanian sites were very close together (less than half a kilometer apart), and yet Jordanian WmCSV sequences showed more within-site genetic variation compared to the more distantly located SLCV sites.

Movement of these viruses over large distances is likely occurring through movement of infected whiteflies and not infected plant material. With the possible exception of exchange between Israel and Palestine, there are strict limitations on the movement of seedlings among these countries. Consequently, differences in migration rates may be due to biological factors affecting whitefly movements within each country, perhaps related to the different time of year when the watermelon crops are planted. However, since both pathogens share the same vector and can coinfect the same cucurbit hosts, it is difficult to imagine the additional hurdles WmCSV faces when dispersing compared to SLCV.

## Conclusions

Our study of emergent cucurbit-infecting begomovirus diversity shows that these pathogens frequently migrate between Middle Eastern nations. This bolsters observations in the field, where skipping a planting season does not diminish the presence of the virus in the next year – the pathogens merely move back in whenever the crop becomes available [13]. Without a greater understanding of the factors that lead to the reduced biogeography of WmCSV, we predict that WmSCV will expand its range to Egypt in the coming years.

## Methods

Symptomatic watermelons (*Citrullus lanatus*) were sampled for WmCSV in June 2011 and squash (*Cucurbita pepo*) were sampled for SLCV in mid September 2011. These times were chosen to correspond to previously observed symptoms in each crop. In each case, approximately 0.1 g of tissue was collected, from the tip of symptomatic watermelon vines and from the 4th leaf from the top of symptomatic squash plants.

Sampling sites were in Egypt (EG), Israel (IL), Jordan (JO), Lebanon (LB) and the regions of the Palestine governed by the Palestinian Authority (PA). In each country, sampling was attempted in up to four separated sites, up to 20 samples per site. Not all of these samples yielded a fully sequenced DNA-A component of a begomoviral genome. Additional collections from 2010 (June for WmCSV, and September for SLCV) were processed for IL and JO.

Agricultural practices differ among the sampled countries, and in some cases crops were grown from seed, some from seedling, and in the case of watermelons, are grown by grafting in Israel and sometimes by grafting in the other countries. Sampled fields within a country did not always have the same cropping practices.

### Sample preparation and sequencing

Total nucleic acids were extracted from leaf samples according to Dellaporta et al. [27]. Samples were tested initially for the presence of SLCV by PCR using primer pair SLCVPL (5′-CCAGGAGGT GTCCTCTCAAC-3′, nucleotides 53 to 72) and SLCVPR (5′-AGAGCGTGAGACCTTTGAGG-3′, nucleotides 444 to 425), which amplifies a 391 bp fragment, and for WmCSV using the primer pair WmCSVPL (5′- TTTCGATACATGGGCCTGTT -3′, nucleotides 49 to 69) and WmCSVPR (5′- TAGCTGGAAATGGGGTTTTG -3′, nucleotides 399 to 379), which amplifies a 350 bp fragment. All amplicons included the common region of their respective DNA-A components. Amplicons were sequenced and compared with DNA-A sequences of SLCV and WmCSV. Plants from which amplicons with a 95% or greater nucleic acid sequence identity to SLCV were considered infected with SLCV, and those with 95% or greater nucleic acid sequence identity to WmCSV were considered infected with WmCSV.

Total nucleic acid extracts of SLCV-infected plants were amplified with two primer pairs, each amplifying approximately half of the viral DNA-A to obtain the full sequence of SLCV DNA-A. Primer pair Xho-SLCV-A-F (5′-CATGATTCTCGAGTACATAATTTAC-3′) and SLCVA2314R (5′- CTGCCTCATTCAATTATCTG-3′) was used to amplify a 1300 bp fragment of SLCV DNA-A and primer pair, SLCVA2295F (5′- CAGATAATTGAATGAGGCAG-3′) and Xho-SLCV-A-R (5′-TGTACTCGAGAATCATGAAATAAAATTC-3′), were used to amplify a fragment of 1500 bp of SLCV DNA-A.

Total nucleic acid extracts of WmCSV-infected plants were amplified with two primer pairs, each amplifying approximately half of the viral DNA-A to obtain the full sequence of WmCSV DNA-A. Primer pair WmA150F (5′- GTCAGTATGTGGGATCCATTGC-3′) and WmA1350R (5′- GCAAATACGATTCAACCACAACC -3′) was used to amplify a 1200 bp fragment of WmCSV DNA-A . Primer pair WmA1325F (5′- GGTTGTGGTTGAATCGTATTTGC-3′) and WmA170R (5′- GCAATGGATCCCACATACTGAC-3′) was used to amplify a 1650 bp fragment of WmCSV DNA-A.

All amplified viral DNAs were cloned into pTZ57R plasmid (Thermo Fisher Scientific, Waltham, MA), and sequenced. The two amplified halves from each infected plant were combined to make a single sequence. This method does potentially create artificial chimeric sequences, but we did not see any evidence of recombination corresponding to these halves in our results (Table 4). Sequences were deposited into GenBank with details about their isolation (SLCV:KM595091-KM595239; WmCSV: KM820183-KM820288).

### Sequence analysis

Treating each species as a separate dataset, whole genome (DNA-A) sequences were aligned manually in Se-Al v2.0a11, using the nick site in the invariant nonanucleotide origin of replication as the site of linearization. Sequences with unique indels that caused frameshift mutations in the CP and Rep were conservatively eliminated from analysis as presumptive amplification and/or sequencing errors. Similarly, sequences with unique premature stop codons in CP and Rep were usually excluded on the same grounds. There were cases where the premature stop codon was fairly close to the end of the full-length protein, and where more than one sequence from the same country contained the identical premature stop codons. In these instances, the sequences remained in the dataset.

The resulting datasets were analyzed for recombination with RDP 3.44a using default settings, except for Kimura 2 parameter nucleotide substitution models instead of Jukes-Cantor where possible [28]. Sequences were considered recombinant if at least three algorithms (of seven: RDP, GENECONV, Bootscan, MaxChi, Chimaera, SiScan, 3Seq) showed statistical support using a Bonferroni-corrected p-value.

Each species’ dataset was analyzed for pairwise nucleotide diversity (π) and divergence among populations (Dxy, Nei 1987; Wright’s F_ST_, [29]) using DnaSP [30]. We examined the phylogenetic relationship of the SLCV and WmCSV isolates with and without previously characterized sequences in GenBank. Appropriate nucleotide substitution models for each dataset were selected using the hierarchical likelihood ratio test in Modeltest [31]. These were used to create Maximum Likelihood (ML) trees using a tree-bisection-reconnection approach in PAUP* [32]. ML trees were bootstrapped 1000 times using nearest neighbor interchange. ML trees were also constructed and were rapid bootstrapped in RaxML 7.2 [33, 34] on the CIPRES server (http://www.phylo.org) assuming the general-time-reversible nucleotide model and a gamma distribution of multiple substitutions. Trees were visualized and edited using FigTree (http://tree.bio.ed.ac.uk/software/figtree) and Adobe Illustrator. Hypothesis testing on these trees using the Shimidaro-Hasegawa test was conducted using RaxML and CONSEL [35].

Pairwise distance matrices of each viral dataset were generated in MEGA 5.22 [36] assuming a Kimura 2 parameter model. Geographic distances between sampling sites were calculated using the great circle distance method as implemented by a National Oceanic and Atmospheric Administration applet (http://www.nhc.noaa.gov/gccalc.shtml). Perl scripts (available upon request) generated a symmetric matrix of distances between isolation sites to match each genetic distance matrix. The relationship between genetic and geographic distance was correlated with a Mantel test in PASSaGE v2 [37], using 999 permutations (α = 0.05).

## References

[1] Jones RA (2009). Plant virus emergence and evolution: origins, new encounter scenarios, factors driving emergence, effects of changing world conditions, and prospects for control. Virus Res.

[2] Duffy S, Seah YM (2010). 98% identical, 100% wrong: per cent nucleotide identity can lead plant virus epidemiology astray. Philos Trans R Soc Lond B Biol Sci.

[3] Duffy S, Holmes EC (2007). Multiple introductions of the Old World begomovirus Tomato yellow leaf curl virus into the New World. Appl Environ Microbiol.

[4] Lazarowitz SG (1991). Molecular characterization of two bipartite geminiviruses causing squash leaf curl disease: role of viral replication and movement functions in determining host range. Virology.

[5] Antignus Y, Lachman O, Pearlsman M, Omer S, Yunis H, Messika Y, Uko O, Koren A (2003). Squash leaf curl geminivirus – a new illegal immigrant from the western hemisphere and a threat to cucurbit crops in Israel. Phytoparasitica.

[6] Abudy A, Sufrin-Ringwald T, Dayan-Glick C, Guenoune-Gelbart D, Livneh O, Zaccai M, Lapidot M (2010). Watermelon chlorotic stunt and Squash leaf curl begomoviruses – New threats to cucurbit crops in the Middle East. Isr J Plant Sci.

[7] Walkey DGA, Alhubaishi AA, Webb MJW (1990). Plant virus diseases in the Yemen Arab Republic. Trop Pest Manag.

[8] Idris AM, Abdel-Salam A, Brown JK (2006). Introduction of the New World Squash leaf curl virus to Squash (Cucurbita pepo) in Egypt: A potential threat to important food crops. Plant Dis.

[9] Al-Musa A, Anfoka G, Misbeh S, Abhary M, Ahmad FH (2008). Detection and molecular characterization of Squash leaf curl virus (SLCV) in Jordan. J Phytopathol.

[10] Sobh H, Samsatly J, Jawhari M, Najjar C, Haidar A, Abou-Jawdah Y (2012). First report of Squash leaf curl virus in cucurbits in Lebanon. Plant Dis.

[11] Ali-Shtayeh MS, Jamous RM, Husein EY, Alkhader MY (2010). First report of Squash leaf curl virus in Squash (Cucurbia pepo), Melon (Cucumis melo) and Cucumber (Cucumis sativa) in the Northern West Bank of the Palestinian Authority. Plant Dis.

[12] Ali-Shtayeh MS, Jamous RM, Hussein EY, Mallah OB, Abu-Zeitoun SY (2014). Squash leaf curl virus (SLCV): a serious disease threatening cucurbits production in Palestine. Virus Genes.

[13] Samsatly J, Sobh H, Jawhari M, Najjar C, Haidar A, Abou-Jawdah Y (2012). First report of Watermelon chlorotic stunt virus in cucurbits in Lebanon. Plant Dis.

[14] Ali-Shtayeh MS, Jamous RM, Hussein EY, Mallah OB, Abu-Zaitoun SY (2012). First report of Watermelon chlorotic stunt virus in Watermelon in the Palestinian Authority. Plant Dis.

[15] Al-Musa A, Anfoka G, Al-Abdulat A, Misbeh S, Haj Ahmed F, Otri I (2011). Watermelon chlorotic stunt virus (WmCSV): a serious disease threatening watermelon production in Jordan. Virus Genes.

[16] Sufrin-Ringwald T, Lapidot M (2011). Characterization of a synergistic interaction between two cucurbit-infecting begomoviruses: Squash leaf curl virus and Watermelon chlorotic stunt virus. Phytopathology.

[17] Khan AJ, Akhtar S, Briddon RW, Ammara U, Al-Matrooshi AM, Mansoor S (2012). Complete nucleotide sequence of watermelon chlorotic stunt virus originating from Oman. Viruses.

[18] Kheyr-Pour A, Bananej K, Dafalla GA, Caciagli P, Noris E, Ahoonmanesh A, Lecoq H, Gronenborn B (2000). Watermelon chlorotic stunt virus from the Sudan and Iran: Sequence Comparisons and Identification of a Whitefly-Transmission Determinant. Phytopathology.

[19] Fletcher J, Luster D, Bostock R, Burans J, Cardwell K, Gottwald T, McDaniel L, Royer M, Smith K, Scheld WM, Grayson ML, Hughes JM (2010). Emerging infectious plant disease. Emerging Infections 9.

[20] Lefeuvre P, Martin DP, Harkins G, Lemey P, Gray AJ, Meredith S, Lakay F, Monjane A, Lett JM, Varsani A, Heydarnejad J (2010). The spread of tomato yellow leaf curl virus from the Middle East to the world. PLoS Pathog.

[21] Lima AT, Sobrinho RR, Gonzalez-Aguilera J, Rocha CS, Silva SJ, Xavier CA, Silva FN, Duffy S, Zerbini FM (2013). Synonymous site variation due to recombination explains higher genetic variability in begomovirus populations infecting non-cultivated hosts. J Gen Virol.

[22] Silva SJC, Castillo-Urquiza GP, Hora-Junior BT, Assunção IP, Lima GSA, Pio-Ribeiro G, Mizubuti ESG, Zerbini FM (2012). Species diversity, phylogeny and genetic variability of begomovirus populations infecting leguminous weeds in northeastern Brazil. Plant Pathol.

[23] García-Arenal F, Fraile A, Malpica JM (2001). Variability and genetic structure of plant virus populations. Annu Rev Phytopathol.

[24] Kassem MA, Juarez M, Gomez P, Mengual CM, Sempere RN, Plaza M, Elena SF, Moreno A, Fereres A, Aranda MA (2013). Genetic diversity and potential vectors and reservoirs of Cucurbit aphid-borne yellows virus in southeastern Spain. Phytopathology.

[25] Ge LM, Zhang JT, Zhou XP, Li HY (2007). Genetic structure and population variability of Tomato yellow leaf curl China virus. J Virol.

[26] Briddon RW, Markham PG (2001). Complementation of bipartite begomovirus movement functions by topocuviruses and curtoviruses. Arch Virol.

[27] Dellaporta SL, Wood J, Hicks JB (1983). A plant DNA minipreparation: version II. Plant Mol Biol Rep.

[28] Martin DP, Lemey P, Lott M, Moulton V, Posada D, Lefeuvre P (2010). RDP3: a flexible and fast computer program for analyzing recombination. Bioinformatics.

[29] Hudson RR, Slatkin M, Maddison WP (1992). Estimation of levels of gene flow from DNA sequence data. Genetics.

[30] Rozas J, Sanchez-DelBarrio JC, Messeguer X, Rozas R (2003). DnaSP, DNA polymorphism analyses by the coalescent and other methods. Bioinformatics.

[31] Posada D, Crandall KA (1998). MODELTEST: testing the model of DNA substitution. Bioinformatics.

[32] Swofford DL (2003). PAUP* Phylogenetic analysis using parsimony (*and other methods).

[33] Stamatakis A, Hoover P, Rougemont J (2008). A rapid bootstrap algorithm for the RAxML Web servers. Syst Biol.

[34] Stamatakis A, Ludwig T, Meier H (2005). RAxML-III: a fast program for maximum likelihood-based inference of large phylogenetic trees. Bioinformatics.

[35] Shimodaira H, Hasegawa M (2001). CONSEL: for assessing the confidence of phylogenetic tree selection. Bioinformatics.

[36] Tamura K, Peterson D, Peterson N, Stecher G, Nei M, Kumar S (2011). MEGA5: molecular evolutionary genetics analysis using maximum likelihood, evolutionary distance, and maximum parsimony methods. Mol Biol Evol.

[37] Rosenberg MS, Anderson CD (2011). PASSaGE: Pattern Analysis, Spatial Statistics and Geographic Exegesis Version 2. Methods Ecol Evol.

